# Effects of Sport-Based Exercise Interventions on Executive Function in Older Adults: A Systematic Review and Meta-Analysis

**DOI:** 10.3390/ijerph191912573

**Published:** 2022-10-01

**Authors:** Falonn Contreras-Osorio, Rodrigo Ramirez-Campillo, Enrique Cerda-Vega, Rodrigo Campos-Jara, Cristian Martínez-Salazar, Rodrigo Araneda, Daniela Ebner-Karestinos, Cristián Arellano-Roco, Christian Campos-Jara

**Affiliations:** 1 Exercise and Rehabilitation Sciences Institute, Faculty of Rehabilitation Sciences, Universidad Andres Bello, Santiago 7591538, Chile; 2 Exercise and Rehabilitation Sciences Institute, School of Physical Therapy, Faculty of Rehabilitation Sciences, Universidad Andres Bello, Santiago 7591538, Chile; 3 Pedagogy in Physical Education and Health Career, Department of Health Science, Faculty of Medicine, Pontificia Universidad Católica de Chile, Santiago 7820436, Chile; 4 Hospital Mauricio Heyermann, Servicio de Psiquiatría, Angol 4650207, Chile; 5 Departamento de Educación Física, Deportes y Recreación, Pedagogía en Educación Física, Facultad de Educación y Ciencias Sociales y Humanidades, Universidad de La Frontera, Temuco 4780000, Chile

**Keywords:** executive function, inhibitory control, working memory, cognitive flexibility, sport, older adults

## Abstract

Exercise programs of moderate-to-vigorous intensity have been shown to improve the cognitive performance of older people. However, the specific effects of sports-based exercise programs on cognitive performance, particularly executive functions, remain unclear. Therefore, the purpose of this study is to clarify the effects of sports-based exercise programs on executive functions in older adults using a systematic review and meta-analysis of the scientific literature. A systematic review was conducted between 1 March and 1 July 2022, to look for published scientific evidence that analyzed different sports programs that may have affected executive function in healthy older adults. Longitudinal studies, which assessed the effects of sports interventions on healthy older adults, were identified through a systematic search of the four principal electronic databases: Web of Science, PubMed, Scopus, and EBSCO. A total of nine studies with a total of 398 subjects met the inclusion criteria and were classified based on one or more of the following categories: working memory, inhibition, and cognitive flexibility. The DerSimonian and Laird random-effects model was performed using the Comprehensive Meta-Analysis software to facilitate the analysis of the studies. Statistical significance was set at *p* ≤ 0.05. In terms of working memory, a small but positive significant effect was noted for the intervention group compared to the control group (effect size (ES) = 0.35, 95% confidence interval (CI) = 0.04–0.67; *p* = 0.029; I^2^ = 36.7%); in terms of inhibition, the intervention had a small favoring but no significant effect compared to the control group (ES = 0.20, 95% CI = −0.42–0.84; *p* = 0.517; I^2^ = 78.8%); and in terms of cognitive flexibility, the intervention had a small favoring but no significant effect compared to the control group (ES = 0.39, 95% CI = −0.11–0.89; *p* = 0.131; I^2^ = 75.5%). Our findings suggest that healthy older adults should be encouraged to participate in sports to improve their working memory; however, more studies are required in this area to reach more robust conclusions. This systematic review was registered with the International Prospective Register of Systematic Reviews (registration number: CRD42022284788).

## 1. Background

Executive functions are a set of mental processes that are responsible for the monitoring and controlling of the mechanisms that mediate information use and allow for the regulation of thoughts and actions during goal-directed behavior [1]. There is a consensus for its classification into three central dimensions: inhibition, working memory, and cognitive flexibility [2,3]. Inhibition allows us to control our attention, behavior, thoughts, and/or emotions to overcome a strong internal tendency to follow impulses, give conditioned responses, or act in response to environmental stimuli while resisting distraction or interference by irrelevant information from the environment or memory [1,4]. Working memory allows us to keep a small amount of information accessible in order to perform cognitive operations, such as manipulating verbal or non-verbal (visual–spatial) content [5], whereas cognitive flexibility refers to our ability to efficiently adjust our behavior in response to changes in the environment [6]. These skills are important for many aspects of daily life in older people, including mental and physical health, social development, function maintenance, and autonomy [7,8,9].

From an early age, executive functions are associated with physical activity [10,11,12,13,14], especially with the practice of sports [15,16,17,18,19]. Such a relationship has also been evidenced in older people, and associations have been reported between complex motor tasks and executive functioning [20]. Some studies suggest that older adults use executive functions to support the execution of complex motor tasks because motor control may become less automated with age [21,22,23]. It must be considered, however, that both cognitive and motor skills experience changes during this stage of life [24,25], making it necessary to implement strategies that allow for coping with the decline in normal functions [26,27].

Physical exercise has been described as an intervention capable of providing cognitive benefits to older adults in order to maintain brain health [28,29,30,31,32]. Evidence suggests that chronic (spreading over weeks, months, or years), moderate-to-vigorous intensity (50–75% heart rate reserve) [33] exercise programs elicit favorable effects on cognitive performance in cognitively normal older adults [34]; this can vary based on aspects such as sex and on the cognitive modality assessed, with larger effects being observed in those measures associated with the executive function and in studies with a higher percentage of women [34,35,36,37]. However, the moderating effect of sex has not been seen in other investigations of the effects of physical training on executive function and possible underlying moderators in cognitively normal older adults and older adults with mild cognitive impairment [38,39]. The mechanisms that have been described to mediate this effect include the influence of prolonged exercise on the expression of neurotransmitters, neurotrophic factors, synaptic plasticity, modification of inflammatory pathways, and cerebrovascular function [34,40,41].

The types of exercise usually employed in chronic intervention programs for the elderly include aerobics, resistance training, mind–body exercise, and multimodal exercise (combining aerobic exercise, resistance training, etc.), and there are systematic reviews and meta-analyses that have attempted to determine the specific effects of these modalities on executive functions [39,42,43]. In this regard, Chen et al. [38] demonstrated that the type of exercise can act as a moderator of the effect observed on the executive functions of older individuals, considering exercise categories such as Tai Chi and yoga (g = 0.38, *p* < 0.05), resistance exercise (g = 0.22, *p* < 0.05), aerobic exercise (g = 0.14, *p* < 0.05), and combined exercise (g = 0.10, *p* < 0.05). Xiong et al. [43] reported that more than 13 weeks of aerobic exercise significantly improves working memory performance and cognitive flexibility in cognitively healthy older adults, while interventions longer than 26 weeks significantly improve inhibition. Training programs with mind–body exercises have also been shown to improve performance in working memory and cognitive flexibility in response to a higher frequency of group practices and additional practice at home (more or equal to five times per week) [39,43]. However, there are several limitations mentioned in previous reviews and meta-analyses that should be considered in future investigations. They include the search’s limitations (in terms of the year of publication of the included studies or language) and the consideration of a limited number of measures for the main dimensions of the executive functions (working memory, inhibition, and cognitive flexibility), which can generate a potential bias on the results obtained [38,42].

In terms of its benefits on executive function in older people, a type of intervention that has not been specifically assessed in previous reviews thus far are those that are structured upon a sport modality, such as combat sports (taekwondo or karate) [44,45,46,47,48], individual sports (swimming, table tennis, golf, or Nordic walking) [49,50,51,52,53], or adapted team sports (walking football) [54]. Pacheco et al. [44] investigated the effectiveness of Karate-Do training on cognition in healthy older adults and identified significant improvements in visual memory and cognitive flexibility after a 12-week intervention period. In contrast, Cho and Roh [45] studied the effects of regular taekwondo training for 16 weeks on physical fitness, neurotrophic growth factors, cerebral blood flow velocity, and cognitive function in healthy elderly women and found a significant improvement in inhibitory control performance after the intervention period. This could be due to increased levels of neurotrophic growth factor.

While physical activity involves movements producing an increase in energy expenditure (due to increased skeletal muscle activity) above resting conditions, exercise (physical exercise) usually entails specific movement patterns performed systematically over a planned schedule to achieve a desired aim in line with improvement in fitness and health-related outcomes [55]. Regarding sport-based activities, these involve movements with defined goals, containing explicit formal rules and structured relationships between participants–athletes [56]. Sport is particularly fundamental as it meets a series of conditions that allow for the enhancement of the executive functions of those who practice it, incorporating complex, controlled, and varied movements, which in turn demand various degrees of adaptation to the environmental requirements [57,58,59,60]. In addition, sports learning is attractive and favors the progressive achievement of aims, thereby promoting emotional and social development, especially when its practice involves contact with other people or being part of a team [61,62,63,64]. The aforementioned aspects could bring sport closer to simultaneous exercise–cognitive training, considering that studies analyzing the effects of aerobic training combined with cognitive challenges are associated with cognitive improvements in healthy older adults, especially regarding executive function, which can prolong functional autonomy among older adults [64,65,66].

Thus, this study aims to systematically collect the available scientific evidence and analyze the effects of sports interventions on the main dimensions of executive functions in healthy older adults compared to their control peers.

## 2. Materials and Analysis

### 2.1. Review Question

What are the effects of sports-based interventions on the main dimensions of the executive functions of healthy older adults compared with active or passive control conditions?

### 2.2. Search Strategy

**Figure 1:**
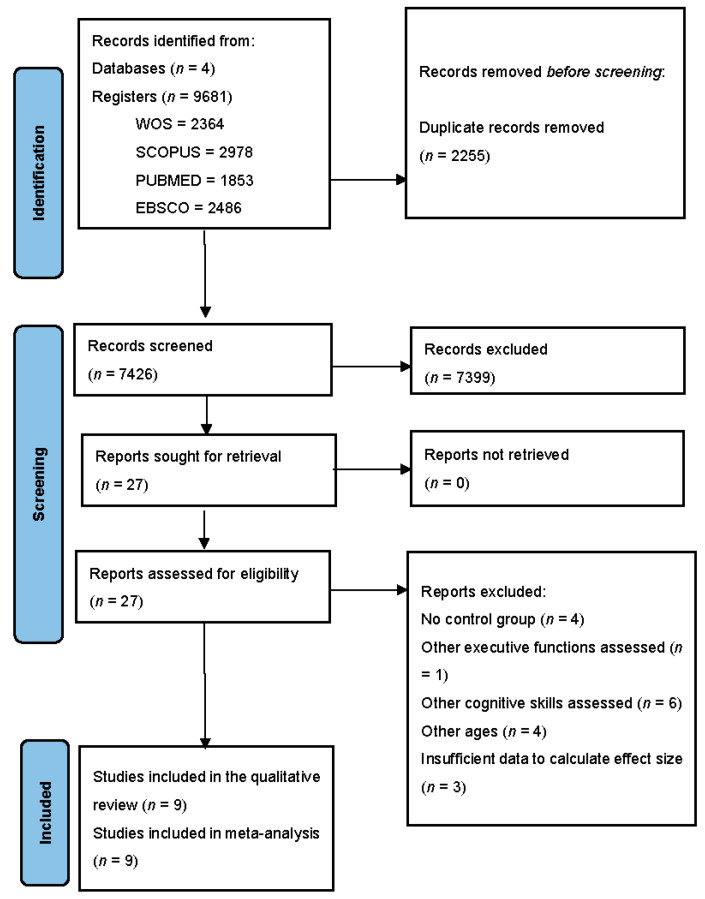
A flow diagram of the studies included in the meta-analysis.

This review was conducted between 1 March and 1 July 2022, according to the guidelines established by Preferred Reporting Items for Systematic Reviews and Meta-Analysis [67] (Figure 1). We pre-registered our meta-analytic review in the International Prospective Register of Systematic Reviews (CRD42022284788).

The following electronic databases were used: Web of Science, PubMed, Scopus, and EBSCO. We combined different keywords and medical subject headings to identify and evaluate relevant studies from inception until June 2022. No filters or limits (for example, regarding language or date of publication) were used to conduct the search. See Supplementary Table S1 for the specific search strategy in each database.

Additionally, reference lists of previous reviews and all included trials were hand-searched to identify potentially eligible studies. Systematic reviews were searched in the same databases with the filters (or terms) “systematic review” OR “reviews” after the regular search strategy. We also consulted two external experts in executive functions (with PhDs and with publications in indexed journals) to examine the inclusion list of articles and to identify possible articles missing from the list. The experts were identified and included based on their Expertscape rank for “Executive + function,” which can be found at https://www.expertscape.com/ex/executive+function (accessed on 16 May 2022).

### 2.3. Eligibility Criteria

Table 1 summarizes the eligibility criteria that were defined based on participants, interventions, comparators, outcomes, and study design (PICOS).

Studies that have been published as original articles in peer-reviewed journals were selected. The studies were available in full text and contained sufficient data to calculate the effect size (ES).

Eligible studies reported pre- and postintervention changes in one or more measures associated with the executive functions of working memory, inhibition, or cognitive flexibility. These tasks were directly applied to participants using instruments validated for the respective population. Some examples of tasks included in the studies are the N-back task [68] to assess working memory, the Stroop task [69] to assess inhibition, or the Trail Making Test-Part B [70] to assess cognitive flexibility.

### 2.4. Data Management

Articles were imported into a reference management system, and duplicates were removed.

Two independent authors (FCO and CCJ) examined the titles and abstracts of the articles found in the databases, using “yes” and “no” instructions to apply the inclusion criteria. If there were disagreements among the authors, the assessment was discussed with a third author (RRC), who worked together to reach a consensus.

The bibliographies of previous reviews and studies ultimately selected were reviewed using the same process described above to identify potential new studies that met the inclusion criteria.

### 2.5. Data Extraction

Data extraction was completed independently by one reviewer (FCO) and verified by a second reviewer (CCJ) for the following: author, publication year, sample size, study characteristics, participants (sex, age, years of schooling, and sports experience), description of the sports training program (structure or stages), sport, intervention length, weekly frequency, session length and intensity, control condition, dimensions of executive function evaluated, tasks completed, reliability indices reported, and a description of the measurement protocol used in each study.

The averages and standard deviations of the dependent variables were extracted before and after the sports interventions in the included studies using Microsoft Excel (Microsoft Corporation, Redmond, WA, USA). When the requested data were not communicated clearly or completely, the authors were contacted to obtain clarifications. When the authors’ responses were not obtained (after several attempts over a 2-week period), the results of these studies were excluded from the analysis. The authors (FCO and CCJ) extracted data independently, and any discrepancies between them (e.g., mean value for a given outcome, number of participants in a group) were resolved through consensus with a third author (RRC).

### 2.6. Risk of Bias (Quality) Assessment

The Physiotherapy Evidence Database (PEDro) scale was used to assess the methodological quality of the included studies, which were rated from 0 (lowest quality) to 10 (highest quality). The validity and reliability of the PEDro scale have been established previously [71,72,73]. Its items primarily assess factors related to the risk of bias in studies. Accordingly, it facilitates comparisons between meta-analyses. The methodological quality of studies was interpreted using the following convention [74,75,76]: ≤3 points represented “poor” quality, 4–5 points represented “moderate” quality, and 6–10 points represented “high” quality. If trials had been previously rated and listed in the PEDro, the respective scores were used. Two authors (FCO and CCJ) assessed the methodological quality of each included study independently, and any discrepancies between them were resolved via consensus with a third author (RRC).

Regardless of their methodological quality, all studies that fulfilled the inclusion criteria were incorporated into the review.

### 2.7. Strategy for Data Synthesis

Although meta-analyses can be performed with as few as two studies [77], because small sample sizes are common in the sports science literature [78], a meta-analysis was only conducted in the present case when >3 studies were available [79,80]. ESs (i.e., Hedges’ g) were calculated for executive function globally and for each dimension of executive function in the experimental and control/comparator groups using the mean and standard deviation before and after the intervention period. If studies reported data other than mean and/or standard deviation values, appropriate statistical conversion was performed before meta-analysis. Data were standardized using postintervention standard deviation values. The DerSimonian and Laird random-effects model was used to account for differences between studies that might affect the intervention effect [81]. The ES values are presented with 95% confidence intervals (95% CIs). Calculated ESs were interpreted based on the following scale: <0.2, trivial; 0.2–0.6, small; >0.6–1.2, moderate; >1.2–2.0, large; >2.0–4.0, very large; and >4.0, extremely large [82]. The level of heterogeneity was assessed using the I^2^ statistic, with values of <25%, 25–75%, and >75% representing low, moderate, and high levels of heterogeneity, respectively [83]. The risk of publication bias was explored for continuous variables (≥10 studies per outcome) [84,85] using the extended Egger’s test [84]. To adjust for publication bias, a sensitivity analysis was conducted using the trim and fill method [86], with L0 as the default estimator for the number of missing studies [87]. All analyses were conducted using the Comprehensive Meta-Analysis software (version 2, Biostat, Englewood, NJ, USA). Statistical significance was set at *p* ≤ 0.05.

## 3. Results

Figure 1 depicts the flow diagram of this article’s search and selection, from systematic search to inclusion.

### 3.1. Study Selection

The electronic search process identified 9681 studies (2364 from WOS; 2978 from SCOPUS; 1853 from PUBMED; and 2486 from EBSCO). Duplicate studies were eliminated (*n* = 2255), the titles and summaries of the remaining studies were reviewed, and another 7399 studies were eliminated. Next, the full text versions of 27 studies were reviewed, and 15 were rejected because they did not include a control group (*n* = 4), included the recruitment of participants of other ages (*n* = 4), evaluated other dimensions of executive functions (*n* = 1), or evaluated other cognitive skills (*n* = 6). Subsequently, data were requested from the authors of five studies because they were insufficient to calculate the ES, and three of them were eliminated because of lack of response. This systematic review and meta-analysis included the nine remaining studies [44,45,46,48,49,50,51,54,88], in which working memory was analyzed in seven studies [44,46,48,49,50,54,88], inhibition in five [45,48,49,54,88], and cognitive flexibility in five [44,49,50,51,88]. The studies included considered nine experimental groups that corresponded to 394 participants, while nine control groups included 337 participants, with a chronological age ranging from 60.3 to 82.7 years. Seven of the nine studies included both men and women, while one included men alone [50] and another included women alone [45]. Table 2 shows the details of the participants’ characteristics.

### 3.2. Study Characteristics

The intervention programs consisted of a sports program, which included one of the following sports: swimming, table tennis, walking football, taekwondo, golf, Karate-Do, and cycling. The total duration of the programs ranged from 8 weeks to 6 months, with 1–5 sessions per week and 40–120 min per session. The control conditions reported were active and passive, considering stretching programs, balance and stretching programs, a health education program, the absence of exercise practice, and the maintenance of daily life activities. The intensity of the intervention program alone was reported in three out of nine studies, with a maximum heart rate of 40–80%. Table 3 depicts a detailed description of these, and other aspects associated with the sports programs.

### 3.3. Methodological Quality

Table 4 shows the results of the methodological quality assessment using the PEDro scale for the nine included studies. In total, three studies received 5 points and were classified as “moderate” in quality, while six studies received 6–10 points and were thus classified as “high” in methodological quality.

### 3.4. Meta-Analysis Results of the Effects of Sport-Based Interventions on Working Memory

**Figure 2:**
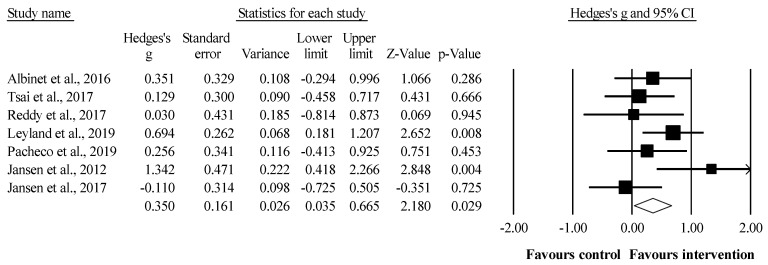
Forest plot of effects on working memory. Values shown are effect sizes (Hedges’s g) with 95% confidence intervals. The size of the plotted squares reflects the statistical weight of the study [44,46,48,49,50,54,88].

The meta-analysis for working memory involved seven studies, seven experimental groups (*n* = 139), and seven control groups (*n* = 116). Compared to the control group, a small but favoring significant effect was observed in the intervention group (ES = 0.35, 95% CI = 0.04–0.67; *p* = 0.029; I^2^ = 36.7%). A sensitivity analysis (each study removed from the analysis once) indicated that the exclusion of three studies [44,49,88] affected the results of the meta-analysis (ES = 0.27–0.37; 95% CI = −0.08–0.74; *p* = 0.052–0.127; I^2^ = 30.6–47.3%) (Figure 2).

### 3.5. Meta-Analysis Results of the Effects of Sport-Based Interventions on Inhibition

**Figure 3:**
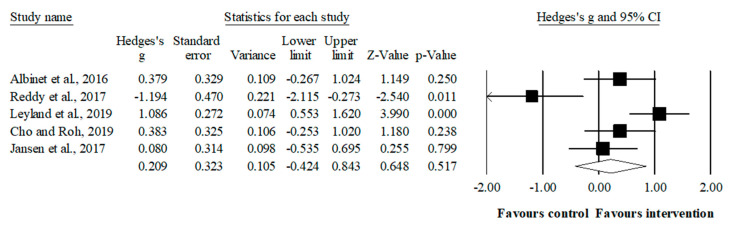
Forest plot of effects on inhibition. Values shown are effect sizes (Hedges’s g) with 95% confidence intervals. The size of the plotted squares reflects the statistical weight of the study [45,48,49,54,88].

The meta-analysis for inhibition involved five studies, five experimental groups (*n* = 108), and five control groups (*n* = 87). Compared to the control group, the intervention had a small favoring but no significant effect (ES = 0.20, 95% CI = −0.42–0.84; *p* = 0.517; I^2^ = 78.8%). A sensitivity analysis (each study removed from the analysis once) indicated that the exclusion of one study [54] affected the results of the meta-analysis (ES = 0.50, 95% CI = 0.05–0.95; *p* = 0.029; I^2^ = 54.9%) (Figure 3).

### 3.6. Meta-Analysis Results of the Effects of Sport-Based Interventions on Cognitive Flexibility

**Figure 4:**
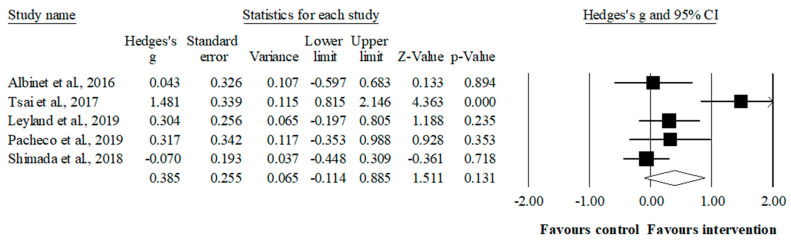
Forest plot of effects on cognitive flexibility. Values shown are effect sizes (Hedges’s g) with 95% confidence intervals. The size of the plotted squares reflects the statistical weight of the study [44,49,50,51,88].

The meta-analysis for flexibility involved five studies, five experimental groups (*n* = 146), and five control groups (*n* = 134). Compared to the control group, the intervention had a small favoring but no significant effect (ES = 0.39, 95% CI = −0.11–0.89; *p* = 0.131; I^2^ = 75.5%). The results remained consistent during the sensitivity analysis (each study removed from the analysis once) (Figure 4).

### 3.7. Adverse Effects

None of the included studies reported soreness, pain, fatigue, injury, damage, or related adverse effects resulting from the sports-based interventions.

## 4. Discussion

In this meta-analysis, we analyzed the results of nine longitudinal studies that included at least one experimental group and one control group as well as pre- and postintervention measurements. The results of our systematic review showed that there are few controlled studies of sufficient methodological quality. The available studies show that sports interventions have a small but favoring significant effect on working memory in healthy older adults when compared to control participants. Furthermore, the effect on inhibition and cognitive flexibility dimensions was not significant.

This systematic review provides innovative results in terms of the type of intervention used, which differs from the physical exercises analyzed in previous studies, which were classified as resistance exercise, aerobic exercise, coordination exercise, multi-component exercise, and mind–body exercise (Tai Chi/Tai Chi Chuan, Qigong, and yoga) [38,39,42,43]. On the contrary, the programs considered in this study were designed according to the original structure of a sport (such as table tennis or golf) or modifications were made to facilitate its practice by older adults (for example, walking football). These modifications allow older people to continue participating in sports while also providing sufficient physiological stimulation to improve their aerobic fitness. This is an effective and safe method of promoting health and well-being [89,90].

This investigation is aimed at highlighting the importance of regular sports practice in old age in order to provide tools for maintaining executive functions and counteracting the normal functional slope that accompanies it [91,92,93]. To achieve this, we analyzed the effects of sports programs on the three main dimensions of executive functions (working memory, inhibition, and cognitive flexibility), excluding indirect or global measures of executive functions because they cannot be compared with the cognitive processes included.

### 4.1. Working Memory

Working memory is an essential system for activities of daily living because it allows for the temporal storage and simultaneous manipulation of information in order to perform complex cognitive tasks in a variety of contexts [5,94]. The results of this meta-analysis indicate that sports-based exercise training improves working memory in older adults, with a small amount favoring a significant effect (ES = 0.35, 95% CI = 0.04 to 0.67; *p* = 0.029; I^2^ = 36.7%) for the intervention group compared to the control group. These results are consistent with a previous meta-analysis conducted by Xiong et al. [43], who assessed the effects of physical exercise interventions, such as aerobic exercise, mind–body exercise, and resistance training, on executive function in cognitively healthy older adults. These investigators showed that physical exercise significantly improves working memory (Hedge’s g = 0.127, 95% CI = 0.052–0.203, *p* < 0.001, I^2^ = 0%). They also detected a significant improvement in aerobic exercise when the exercise mode was examined as a moderator (Hedge’s g = 0.098, 95% CI = 0.017–0.178; *p* = 0.017, I^2^ = 0%). Only three new studies reported information on the intensity of their programs regarding the aerobic component of sports interventions, with a range between 40% and 80% of maximum heart rate, which are considered activities of moderate-to-vigorous intensity [45,49,54]. 

### 4.2. Inhibition

Inhibitory control is the ability to manage attention, behavior, thoughts, and emotions in order to suppress internal or external stimuli that interfere with our ability to choose the best response in a given context [95]. The findings of this meta-analysis showed that the intervention had a small favoring, but non-significant effect compared to the control group (ES = 0.20, 95% CI = −0.42 to 0.84; *p* = 0.517; I^2^ = 78.8%). The sensitivity analysis, however, indicated that the exclusion of a study [54] affected the results of the meta-analysis (ES = 0.50, 95% CI = 0.05 to 0.95; *p* = 0.029; I^2^ = 54.9%). This suggests that the conclusions drawn from this result should be approached with caution in anticipation of future research that will shed light on the effects of sports on inhibition capacity in older adults. The findings of Reddy et al. [54] could be attributed to the exercise dose used, which the authors believe was insufficient (one 60 min session per week), or to the fasting condition under which the cognitive evaluations were performed. Furthermore, no information is provided about the condition of the control as well as the control for the sports experience, physical activity, and medication of participants in this study. Despite these limitations, it should be noted that adapted sports have been shown to be an alternative that promotes an increase in physical condition as well as improvements in anthropometric variables, social contact, and mental health among the elderly. More research in this area would be required to clarify its specific effects on executive functions [89,96].

In the recent review by Ren et al. [39], the effects of Chinese mind–body exercises on the executive function of individuals aged ≥50 years were analyzed and significant results on inhibition capacity were obtained (SMD = 0.18, *p* = 0.107). According to the authors, this could be due to the limited number of studies considered in the research field (13 studies). This factor may also have reduced the possibility of obtaining significant values in our study because the meta-analysis on the inhibition included only five studies. In contrast, the meta-analysis by Xiong et al. [43] identified a positive effect of physical exercise on inhibitory control (Hedge’s g = 0.136; *p* = 0.001, I^2^ = 0%) in cognitively healthy older adults, including a total of 15 studies for this cognitive dimension. The meta-analysis conducted by Wollesen et al. [66] of the effects of cognitive–motor training interventions (including dual-task and technology-based exergame-type interventions) on the executive functions in older people also found a significant positive impact on inhibitory control (mean difference, 0.61; 95% CI, 0.28–0.94; *p* = 0.0003). The increased magnitude of the ES and significance obtained in the above-mentioned meta-analysis can be attributed to the inclusion of studies incorporating a component of cognitive intervention aimed specifically at inhibitory control [97,98,99,100,101]. Apart from the cognitive effort that implies the need to respond to immediate external stimuli (rapid presentation) that arise around us or to unpredictable situations, which promotes the suppression of irrelevant stimuli or responses integrated with physical exercise training in tasks that integrate dancing, walking, balance, and coordination. These components could be addressed in future studies by incorporating activities that focus on self-regulation management or the suppression of irrelevant stimuli as part of sports training for seniors. This has been implemented in sports-based interventions with younger individuals [102,103,104], with reported improvements in executive functions such as inhibitory control.

### 4.3. Cognitive Flexibility

Cognitive flexibility makes it possible to shift attention between multiple tasks based on the demands of the environment or previously established priorities [105]. In terms of the effect reported in this meta-analysis, the intervention had a small favoring, but non-significant effect compared to the control group (ES = 0.39, 95% CI = −0.11 to 0.89; *p* = 0.131; I^2^ = 75.5%). This skill is essential for achieving optimal performance in situations related to sports, especially when the environment and priorities change rapidly [106]. This would lead us to hypothesize that sports-based interventions have a significant positive effect on older adults, as evidenced by a recent review [18] examining the effects of sports-based interventions on children and adolescents. However, one possible explanation for the findings obtained in this study could be related to the characteristics of the intervention programs or the contexts in which they were implemented. They may not have considered the sufficient demand for cognitive flexibility to drive its improvement, or the undeclared exercise intensity may have played a role in four of the five studies included in the meta-analysis of this executive function dimension [44,50,51,88]. Our results are consistent with those of a meta-analysis conducted by Wollesen et al. [66], who found no significant effect of cognitive–motor training interventions on cognitive flexibility. In contrast, Ren et al. [39] and Xiong et al. [43] found evidence of significant positive effects in their respective meta-analyses of mind–body intervention, aerobic exercise, and resistance training programs on executive functioning in older adults. Finally, the lack of significant differences found in cognitive inhibition and flexibility may be partially explained by the daily activities of the control groups, the details of which were not provided, or by the inclusion of studies in which the control groups received other intervention programs such as balance and stretching [49,50].

In terms of the methodological quality of the included studies, none of the analyzed cognitive dimensions received a rating of >7. This suggests that efforts should be made to improve the methodological quality of future studies that address this subject, such as focusing on blinding both subjects and those who administer the therapy. These are flaws in the studies included in our investigation, with which none of them complied satisfactorily. This is a critical factor that can be improved, as highlighted by Wollesen et al. [66], because only 12% of the studies in their review blinded their participants, while 16% blinded the therapists who managed the intervention, and 12% blinded all evaluators. In terms of this latter aspect, our review found that eight of the nine studies included complied satisfactorily with this indicator. On the contrary, Zhidong et al. [42] found a low level of compliance in the blinding of subjects and therapists who manage the intervention in their review. Only 1 of the 29 articles included complied satisfactorily with the first of these aspects, and none of the articles included complied with the second aspect.

### 4.4. Potential Limitations and Suggestions for Future Research

Certain limitations must be considered when interpreting the results: (1) Some of the analyzed studies provided information on relevant variables such as sports experience (*n* = 5), the intensity of sports activities (*n* = 6), and the physical activity levels of their participants (*n* = 3). We suggest that future investigations consider reporting this information in greater detail to facilitate analysis of the effects of their intervention programs. (2) The small number of available studies limits the possibility of conducting additional analyses of potential moderators; however, future investigations could examine the possible differences in the effects of sports-based exercise programs for elderly men and women. (3) Although the investigations included in the specification did not specify any adverse events associated with the sports-based intervention exercises, it is unclear whether the studies did not report them because no cases were registered or whether they simply did not report the information because this aspect was not evaluated. Therefore, we recommend that future studies collect data and describe possible injuries, pain, and/or any other potential adverse effects in detail because this would increase our understanding of the safety of sports-based exercise interventions for older adults.

We suggest further investigations in this area to generate more robust conclusions, considering that the reports are based on a small number of selected studies and that the sensitivity analysis suggests that the exclusion of some studies for both working memory and inhibition affects the results of the meta-analysis as well as the presence of moderate–high heterogeneity (I^2^) values. This would allow for addressing more specific variables, such as the type of sport used in the interventions, considering that the cognitive effects of open- and closed-skill sports may differ [91,107,108,109]. This would be extremely beneficial for expanding the existing body of information as well as providing strategies that adapt to the interests and motivation of elderly people, who can benefit from the process of acquiring new skills or resuming sports activities that they have been unable to do for various reasons throughout their lives.

## 5. Conclusions

Sports-based exercise interventions are more effective in improving measures of working memory compared to control conditions involving active and non-active participants (including alternative training interventions). These findings are derived from nine studies of moderate-to-high methodological quality and a moderate impact of heterogeneity (I^2^). Future studies should clarify the role of sports-based exercise interventions on inhibition and cognitive flexibility owing to the high impact of heterogeneity and limited number of studies.

## Figures and Tables

**Table 1:** Eligibility Criteria Based on PICOS.

PICOS	Inclusion Criteria	Exclusion Criteria
Population	Healthy older adults (mean age of the sample ≥60 years), without restriction according to sex or fitness level.	Children, adolescents, or middle-aged adults. Individuals with a medical condition that may limit their participation in sport-based activities, meaning that they must not have any neurological pathology, psychiatric disorder, or other types of medical conditions. Participants of paralympic sports or individuals with disabilities will not be included.
Intervention	Chronic intervention programs (with a minimum duration of 4 weeks) based on a sport, of a competitive or recreational type. The interventions should involve sport exercises (e.g., soccer) or sport-based or sport-adapted exercises (e.g., walking soccer).	Acute interventions. Chronic sports interventions combined with different types of exercises (for example, aerobics or resistance training) or with the support of a nutritional supplement. Chronic interventions not related to sports.
Comparator	Group not exposed to the sports training program. The control group may be active (alternative training method such as a balance or stretching program) or passive (continuing their usual activities of daily living).	Absence of a control group.
Outcome	Pre-/postintervention values for one or more direct assessment measures for executive functions of working memory, inhibition, or cognitive flexibility.	Indirect measures of executive functions (e.g., questionary). Measures of executive functions other than working memory, inhibition, or cognitive flexibility.
Study design	Longitudinal studies with at least one experimental group and control group, including pre- and postintervention measurements.	Cross-sectional studies; single-group interventions.

**Table 2:** Subjects’ characteristics from the included studies.

References	Country	N	Sex (M/F)	Age (years)	Education (years)	Health Status	Cognitive Status	Level of Physical Activity	Experience with Sports
Albinet et al., 2016 [49]	France	36; EG: 19, CG: 17	10 M/26 F	EG: 67 ± 5, CG: 66 ± 5	EG: 11.89 ± 3.87; CG: 11.59 ± 2.12	Healthy	MMSE EG: 29.11 ± 1.05, CG: 28.74 ± 1.50;	PASD EG: 14.89 ± 4.82, CG: 16.12 ± 4.26	Not reported
Tsai et al., 2017 [50]	China	43; EG: 22, CG: 21	43 M	EG: 66.88 ± 4.74, CG: 65.70 ± 3.54	EG: 12.50 ± 4.09; CG: 10.62 ± 3.20	Healthy	MMSE EG: 28.73 ± 1.28, CG: 27.67 ± 1.80	Sedentary	No regular participation in exercise or sports in the previous 3 months
Reddy et al., 2017 [54]	United Kingdom	20; EG: 11, CG: 9	17 M/3 F	Mean age EG: 61.1; mean age CG: 60.3	Not reported	Healthy	Not reported	Not reported	Not reported
Shimada et al., 2018 [51]	Japan	106; EG: 53, CG: 53	57 M/49 F	EG: 70.1 ± 4.0; CG: 70.7 ± 4.7	EG: 12.8 ± 2.8; CG: 13.7 ± 2.6	Healthy. Subjects with a history of stroke were included, n (%) GE: 5 (9.4), CG: 1 (1.9)	MMSE EG: 28.4 ± 1.8, CG: 28.7 ± 1.4	Sedentary and mild-to-moderate habitual exercise, n (%) EG: 26 (49.1), CG: 26 (49.1)	Those who played golf two or more times per year were excluded
Leyland et al., 2019 [88]	United Kingdom	62; EG: 36, CG: 26	23 M/39 F	EG: 63.03 ± 7.47; CG: 66.04 ± 8.84	EG: 16.83 ± 3.89; CG: 15.94 ± 1.97	Healthy	MMSE EG: 26.86 ± 1.90, CG: 27.58 ± 1.21	PASE (SD) EG: 40.86 (24.84), CG: 35.23 (17.25)	No cycling practice in the last 5 years
Pacheco et al., 2019 [44]	Brazil	33; EG: 16, CG: 17	2 M/ 31 F	EG: 69.06 ± 7.40; CG: 68.35 ± 6.89	EG: 13.88 ± 4.86; CG: 13.00 ± 4.53	Healthy	MMSE EG: 26.94 ± 2.27, CG: 27.47 ± 2.10	They did not exercise regularly.	No previous experience in Karate-Do
Cho and Roh, 2019 [45]	Korea	37; EG: 19, CG: 18	37 F	EG: 68.89 ± 4.16; 69.00 ± 4.41	EG: 11.33 ± 2.47; CG: 11.37 ± 2.41	Healthy	MMSE EG: 26.89 ± 1.81, CG: 26.74 ± 1.63	They did not exercise regularly.	Not reported
Jansen et al., 2012 [46]	Germany	21; EG: 12; CG: 9	5 M/16 F	EG: 73.6 ± 3.9; CG: 82.7/6.6	Not reported	Healthy	Normal	Not reported	Not reported
Jansen et al., 2017 [48]	Germany	40; EG: 23, CG: 17	14 M/25 F One person did not provide information on sex	EG: 62.57 ± 4.19; CG: 65.24 ± 4.66	Not reported	Healthy	Normal	Not reported	Not reported

BDI-II: Beck Depression Inventory, 2nd edition; CG: control group; DSST: digit symbol substitution test; EF: executive function; EG: experimental group; F: female; M: male; Mill Hill: vocabulary test, part B; MMSE: Mini Mental State Examination; N: number of subjects; NCGG-FAT: National Centre for Geriatrics and Gerontology-Functional Assessment Tool; SD: standard deviations; PASD: physical activity score of Dijon; PASE: physical activity for the elderly.

**Table 3:** Intervention characteristics of the included studies.

References	Sport-like Training	Control Condition	Sport	Compliance with the Intervention Program	Length of Intervention	Weekly Frequency	Length of Session	Intensity	EF Tasks
Albinet et al., 2016 [49]	Swimming. Warm-up for 10 min, core session for 40 min, and cool-down for 10 min. The program included activities such as different types of swimming and aquatic fitness exercises.	Stretching	Swimming	EG: 80.3%, CG: 76.5%	21 weeks	2/week	60 min	40–65% HRmax	Stroop task, random number generation task, Hayling task, spatial running span task, verbal running span task, 2-back task, dimension-switching task, plus–minus task, and digit–letter task
Tsai et al., 2017 [50]	Table tennis. Warming up is the most important part of table tennis training, followed by playing games of table tennis with the coach and cooling down. Seven main components over the whole training session were as follows: (a) footwork; (b) serving; (c) forehand and backhand driving; (d) forehand bouncing, backhand bouncing, and alternate bouncing; (e) smashing; (f) continuously hitting back a ball that was randomly delivered by the ball-projection machine from fixed or random directions; and (g) comprehensive practice.	Balance and stretching	Table tennis	90% ± 2%	24 weeks	3/week	40 min	Not reported	Task switching paradigm; N-back task
Reddy et al., 2017 [54]	Walking football. Warm-up, followed by 45–50 min of playing. A small-sided game of walking football, usually five-a-side.	Not reported	Walking football	Each EG participant participated in at least 7 sessions out of 12 (mean, 9.4; mode, 11)	12 weeks	1/week	60 min	76% HRmax; mean Borg RPE: 13.31 (range 9–17 on a scale of 0–20)	Random number generator task
Shimada et al., 2018 [51]	Golf. A total of 14 practice sessions and 10 golf course sessions. (1) Practice sessions: warm-up and stretching exercises of 10 min; sessions 1–9, participants engaged in 70 min of Starting New at Golf training; sessions 7–14, participants practiced at a driving range followed by a 10 min cool-down period. (2) Course sessions: 10 min warm-up and stretching exercises, followed by a half round of golf (100 min) and a 10 min cool-down period.	Health education program	Golf	96.2% attended at least 80% of sessions	24 weeks	Not reported	90–120 min	Not reported	Trail Making Test-Part B
Leyland et al., 2019 [88]	Cycling. Outdoor cycling. Cycling was completed in the Reading and Oxford areas.	No exercise	Cycling	Not applicable because there was no fixed number of scheduled sessions	8 weeks	3/week	30 min	Not reported	Verbal fluency, plus–minus task, letter updating task, Stroop task, stop-it task, and Eriksen flanker task
Pacheco et al., 2019 [44]	Karate-Do. (1) brief warm-up of 5–10 min; (2) kihon exercises, kata (sequences of Karate-Do movements), kumite, and breathing techniques for 40-–45 min; and (3) relaxation through brief meditation exercises tailored to the needs of the participants for 10 min.	Daily activities	Karate-Do	Not reported	12 weeks	2/week	60 min	Not reported	Digit span (backward), verbal phonemic fluency, verbal semantic fluency (animals), Trail Making Test-Part B
Cho and Roh, 2019 [45]	Taekwondo. A total of 10 min of warm-up and cool-down through stretching, and 50 min of main exercise. Main exercise: 5 min of five basic TKD movements (stance, block, punch, strike, and thrust); 10 min of Poom-sae Taegeuk chapter 1–4, 10 min of kicking sessions with basic kicking, steps, and mitt kicks; and 15 min of Taekwon gymnastics.	Daily activities	Taekwondo	Not reported	16 weeks	5/week	60 min	50–80% HRmax	Stroop color and word test
Jansen et al., 2012 [46]	Karate-Do. Training was conducted according to the guidelines of the German Karate Federation. Karate-Do involves powerful movements of the legs or arms (or both at the same time). Long sequences of arm and leg movements were taught. Every training session was led by a professional karate teacher.	Daily activities	Shotokan Karate-Do	Not reported	3–6 months (20 training sessions)	Not reported	60 min	Not reported	Digit backward; Block-tapping test
Jansen et al., 2017 [48]	Karate-Do. Training was conducted according to the guidelines of the German Karate Federation. Participants practiced attacking and defending techniques with a partner. The emphasis was on cooperative training to ensure that both partners benefited from the training. Participants learned simultaneous leg and arm movements as well as training with a partner. In terms of Kata, participants learned the ‘‘Heian Shodan”.	Daily activities	Shotokan Karate-Do	Mean number of completed training sessions 13.26 ± 1.48	8 weeks (15 sessions)	2/week	60 min	Not reported	Stroop color–word interference test; digit backward

CG: control group; EF: executive function; EG: experimental group; HRmax: maximum heart rate; RPE: rate of perceived exertion; SD: standard deviations; TKD: taekwondo.

**Table 4:** PEDro scale of the studies included.

Study	1	2	3	4	5	6	7	8	9	10	11	Total
Albinet et al., 2016 [49]	1	1	0	1	0	0	0	1	0	1	1	5
Tsai et al., 2017 [50]	1	1	0	1	0	0	1	1	0	1	1	6
Reddy et al., 2017 [54]	1	1	1	0	0	0	1	1	0	1	1	6
Shimada et al., 2018 [51]	1	1	0	1	0	0	1	1	0	1	1	6
Leyland et al., 2019 [88]	1	0	0	1	0	0	1	1	0	1	1	5
Pacheco et al., 2019 [44]	1	1	1	1	0	0	1	1	0	1	1	7
Cho and Roh, 2019 [45]	1	1	0	1	0	0	1	1	0	1	1	6
Jansen et al., 2012 [46]	1	0	0	1	0	0	1	1	0	1	1	5
Jansen et al., 2017 [48]	1	1	0	1	0	0	1	1	0	1	1	6

PEDro: Physiotherapy Evidence Database scale.

## Data Availability

Not applicable.

## Supplementary Materials

The following supporting information can be downloaded at: https://www.mdpi.com/article/10.3390/ijerph191912573/s1, Supplementary Table S1: Specific search strategy for each database; Supplementary Data: search strategy and criteria inclusion. References [44,45,46,47,48,49,50,51,52,53,54,88,110] are cited in the Supplementary Materials.
