# Supplementary material for: Effects of Sport-Based Exercise Interventions on Executive Function in Older Adults: A Systematic Review and Meta-Analysis
Source: Int J Environ Res Public Health. 2022 Oct 1;19(19):12573. doi: 10.3390/ijerph191912573 (PMC9566349; doi:10.3390/ijerph191912573)
Supplement: Supplementary file 1 [file ijerph-19-12573-s001.zip › Supplementary Table S1.pdf]

Supplementary Table S1: Specific search strategy for each database.

Database	Specificities of the database	Search strategy
EBSCO	EBSCO does not allow combinations of title and abstract. To avoid multiple internal combinations, we decided to use a more open search strategy in this database, with all code lines being open to "All text".	TX ("executive functions" OR "cognitive functions" OR cognition OR "inhibitory control" OR inhibition OR "working memory" OR shifting OR "cognitive flexibility") AND TX (sports OR "modified sport" OR exercise OR "physical activity" OR athletics OR "sport practice") AND TX ("older adults" OR aging OR elderly OR aged OR "older people")
PubMed	Nothing to report.	(("executive functions"[Title/Abstract] OR "cognitive functions"[Title/Abstract] OR cognition[Title/Abstract] OR "inhibitory control"[Title/Abstract] OR inhibition[Title/Abstract] OR "working memory"[Title/Abstract] OR shifting[Title/Abstract] OR "cognitive flexibility"[Title/Abstract]) AND (sports[Title/Abstract] OR "modified sport"[Title/Abstract] OR exercise[Title/Abstract] OR "physical activity"[Title/Abstract] OR athletics[Title/Abstract] OR "sport practice"[Title/Abstract])) AND ("older adults"[Title/Abstract] OR aging[Title/Abstract] OR elderly[Title/Abstract] OR aged[Title/Abstract] OR "older people"[Title/Abstract])
Scopus	In Scopus, the search for title or abstract also includes keywords.	TITLE-ABS-KEY ("executive functions" OR "cognitive functions" OR cognition OR "inhibitory control" OR inhibition OR "working memory" OR shifting OR "cognitive flexibility") AND TITLE-ABS-KEY (sports OR "modified sport" OR exercise OR "physical activity" OR athletics OR "sport practice") AND TITLE-ABS-KEY ("older adults" OR aging OR elderly OR aged OR "older people")
Web of Science	In Web of Science, the search for title or abstract also includes keywords, and is termed "topic".	((TS=("executive functions" OR "cognitive functions" OR cognition OR "inhibitory control" OR inhibition OR "working memory" OR shifting OR "cognitive flexibility")) AND TS=(sports OR "modified sport" OR exercise OR "physical activity" OR athletics OR "sport practice")) AND TS=("older adults" OR aging OR elderly OR aged OR "older people")<br>https://www.webofscience.com/wos/woscc/summary/1e82b09c-85e7-4c70-92d9-60af5b107fd0-48df71c9/relevance/1 accessed on 26 August 2022
